# Concerns on Generalizability

**DOI:** 10.2196/50280

**Published:** 2023-09-21

**Authors:** Yongjian Lin

**Affiliations:** 1 Department of Gastrointestinal and Gland Surgery First Affiliated Hospital of Guangxi Medical University Nanning China

**Keywords:** mHealth app, mobile health, mHealth, app, prediabetes, traditional Chinese medicine, TCM, health-related quality of life, body constitution, meridian energy

We read the study by Chung et al [1], which evaluated the effectiveness of a mobile health (mHealth) app based on traditional Chinese medicine (TCM) in patients with prediabetes. This randomized controlled trial showed that the TCM mHealth app improved physical energy, fitness, and quality of life in patients with prediabetes. However, the small sample sizes, short follow-up time, and multiple comparisons might limit the generalizability of the findings. After carefully reading this article, we present the suggestions below.

First, as the authors described in the *Methods* section, participants were randomized by a computer-generated randomization list into 3 groups—the TCM mHealth app, the ordinary mHealth app, or the control group—rather than propensity score matching [2]. In addition, most patients have individual polymorphisms in the real world, and although baseline patient characteristics are almost impossible to match in clinical studies (ie, clinical characteristics among patients in this study who received the TCM mobile), there were no significant differences between the general app and control groups. For baseline data, variable transformation, a nonparametric test using rank, or an approximate *t* test could be considered when performing a comparison between 2 small sample means if their overall variances are not equal. Therefore, the results of this study may not truly reflect patients in the real world.

Second, the statistical analysis presents descriptive characteristics as percentages or as mean (SD), as appropriate. A paired *t* test was used to examine the changes in outcome variables within groups and a 1-way ANOVA was used for comparisons among groups. However, there was no description of these data. According to Bridge and Sawilowsky [3], the Wilcoxon rank-sum test is recommended if the population characteristics are unknown, such as yang-deficiency, yin-deficiency, phlegm-stasis, body energy, and physical and mental component scores, and if the hypothesis being tested is a shift in means (or another location parameter).

Third, an interaction is an action that occurs when 2 or more objects interact with each other [4]. Since many variables are described in the first table of Chung et al’s [1] paper, prespecified subgroup analyses based on these variables are necessary. Subgroup analyses based on age, gender, BMI, body composition, and blood pressure were not performed in this study. We suggest that prespecified subgroup analyses be conducted in the TCM mHealth app group, which might allow for more accurate conclusions.

In conclusion, we thank the authors for this excellent work, which provides important evidence for the integration of TCM concepts into an mHealth app for patients with prediabetes. However, we believe that the conclusions of the study would have been stronger if the abovementioned issues had been addressed.

## Abbreviations

mHealth: mobile health
TCM: traditional Chinese medicine
